# Angiographic Safety and Efficacy of the ReSolv Flow-Diverting Stent in a Rabbit Model

**DOI:** 10.1177/15910199241260896

**Published:** 2024-06-20

**Authors:** Rosalie EA Morrish, Alec T Chunta, Brooke L Belanger, Paige M Croney, M Suheel Abdul Salam, Crista Thompson, Muneer Eesa, John H Wong, Alim P Mitha

**Affiliations:** 1 Department of Clinical Neurosciences, 2129 University of Calgary, Calgary, Alberta, Canada; 2 Hotchkiss Brain Institute, 2129 University of Calgary, Calgary, Alberta, Canada; 3 Department of Biomedical Engineering, 2129 University of Calgary, Calgary, Alberta, Canada; 4 Section of Neurosurgery, 8664 University of Manitoba, Winnipeg, Manitoba, Canada; 5 Fluid Biomed, Calgary, Alberta, Canada

**Keywords:** aneurysm, flow-diverting stent, endovascular, occlusion, efficacy, safety

## Abstract

**Background:**

Bioresorbable polymer-based flow-diverting stents have potential benefits over existing metal devices. This study aimed to evaluate the safety and efficacy of the novel ReSolv device, which is a primarily polymer-based flow-diverting stent, using the in vivo rabbit sidewall saccular aneurysm model.

**Methods:**

ReSolv stents were deployed in 14 New Zealand White rabbits that had undergone aneurysm creation procedures. Animals were allocated to follow-up time points of 1, 3, 6, 9, 12, 16, or 18 months. Angiographic images were evaluated by an independent neurointerventionalist blinded to follow-up time points for (1) in-stent stenosis, (2) parent vessel and jailed side branch patency, (3) wall apposition, and (4) aneurysm occlusion using the Raymond-Roy Occlusion Classification (RROC), O’Kelly Marotta grading scale, and the 4F flow diversion predictive score. Primary efficacy outcome was defined as RROC Class I or II.

**Results:**

At a median follow-up time of 7.5 months, parent vessel (14/14) and jailed side (33/33) branches were patent in all cases. There was no development of thrombus on the stent or cases of significant in-stent stenosis, and all stents had good wall apposition. Adequate occlusion was found in 85.7% (*n* = 12) of animals, including an RROC Class I in 64.3% (*n* = 9) and RROC Class II in 21.4% (*n* = 3).

**Conclusions:**

The ReSolv stent shows encouraging angiographic safety and efficacy outcomes after placement in a rabbit sidewall saccular aneurysm model. Longer term studies are ongoing to determine eventual fate of the aneurysm, parent vessel, and jailed side branches after absorption of the polymer component of the stent.

## Introduction

Unruptured intracranial aneurysms (IAs) affect 5% to 10% of the global population.^1^ Current treatments for IAs include open surgical clipping, as well as endovascular procedures such as coiling or flow diversion. Flow-diverting stents (FDSs) are a newer, increasingly popular treatment option that allows the interventionalist to avoid manipulation of devices within the fragile aneurysm sac.^2^

FDSs are metal devices with a high material surface area that bridge the neck of IAs and redirect blood flow away from the aneurysm sac.^3^ This causes stagnation of flow within the aneurysm leading to thrombosis, with eventual endothelialization of the stent across the aneurysm neck resulting in permanent occlusion. The downside of flow diversion is the high thrombogenicity of these devices and the general requirement for lifelong antiplatelet agents, as well as the inability to access the aneurysm sac should the lesion persist. Aneurysm persistence rates after flow diversion are variable depending on the device but are in the range of 25% to 40% at 1 year after treatment.^4,5^ This likely reflects differences in design, including porosity and pore density, as well as stent material and number of layers, leading to differing ability to endothelialize. Furthermore, the information obtained from non-invasive follow-up imaging, such as computed tomographic angiography (CTA) or magnetic resonance angiography (MRA) is limited, often requiring patients to undergo more invasive digital subtraction angiography (DSA) procedures. In order to mitigate some of the challenges associated with aneurysm persistence, and to facilitate re-treatment of the sac if necessary, partially or fully bioresorbable FDSs have been proposed as a potential solution.^6^

The ReSolv stent (Fluid Biomed, Calgary, Canada) is a novel hybrid primarily polymer-based flow diverter with limited metal in its structure that is currently in development. The stent is made from at least 40 bioresorbable poly-l-lactic acid (PLLA) fibers and up to 8 platinum-based radio-opaque strands, and is deployed with a similar technique as metal FDSs.^7^ Initial results have demonstrated a favorable angiogenic safety profile, including mechanical behavior and hemocompatibility.^8^ A pre-clinical study has also shown promising in vitro and in vivo immediate flow diversion characteristics.^7^ However, medium-term occlusion rates after treatment of aneurysms using the ReSolv stent have not yet been reported. The aim of this study, therefore, was to evaluate the angiographic efficacy of the ReSolv stent and to characterize its effect on the parent vessel and jailed side branches in a rabbit sidewall aneurysm model.

## Methods

### Aneurysm model

These studies were performed with approval from the Animal Care and Use Committee of our institution (Protocol AC21-0119). Fifteen (8 female, 7 male) New Zealand White rabbits weighing 2.85 ± 0.35 kg (mean ± SD) at study onset were included in this study. Animals were allocated to the following angiographic follow-up time points: 1 month (*n* = 1, 1 M), 3 months (*n* = 4, 2F and 2 M), 6 months (*n* = 2, 2F), 9 months (*n* = 3, 2F and 1 M), 12 months (*n* = 2, 2 M), 16 months (*n* = 1, 1F), and 18 months (*n* = 2, 1F and 1 M). One animal from the 12-month group was subsequently excluded due to the development of a tracheal branch emanating from the tip of the aneurysm. This study was based on an ongoing longitudinal investigation documenting the absorption of the polymer-based stent over time. Therefore, follow-up and termination time points varied since follow-up angiograms could only be done twice (one at designated follow-up interval, and one at termination).

Prior to surgery, animals were sequentially dosed with acepromazine (0.15 mg/kg IV), isoflurane (1–5% in 100% O_2_), meloxicam (0.4 mg/kg SQ), and buprenorphine (0.03 mg/kg SQ). Rabbits were then intubated and maintained on 2–3% isoflurane in 100% oxygen during the procedure, with continuous monitoring of electrocardiographic data, arterial pressure, oxygen saturation, and temperature.

Sidewall saccular aneurysms were created in all rabbits under sterile conditions based on a previously published method.^9^ Briefly, the right common carotid artery (RCCA) was exposed and temporarily clipped adjacent to the bifurcation of the brachiocephalic artery. Four centimeters from the bifurcation, the RCCA was ligated and a 24-gauge angiocatheter was placed retrograde into the lumen. Roughly 0.6 mL of a 50:50 mixture of elastase (∼4 u/mgP and ∼25 mgP/mL) and 0.5 M CaCl_2_ was infused through the angiocatheter into the isolated segment of the RCCA. After a 20-minute incubation period, the vessel was ligated proximal to the angiocatheter insertion site, the distal section between the two ligatures was cut, and the temporary clip was removed, allowing for reperfusion of the isolated vessel segment. Before closing, the area was irrigated with saline. The incision was then closed in a multilayer fashion with 4-0 vicryl sutures, and animals were monitored during recovery. Two additional doses of buprenorphine and one additional dose of meloxicam were administered for post-procedure analgesia.

### Stent deployment and follow-up angiography

At least 4 weeks after aneurysm creation, the stent deployment surgery was conducted. One week prior to the stent deployment procedure, rabbits were administered ASA (10 mg/kg, PO, daily) indefinitely and clopidogrel (10  mg/kg, PO, daily) for 6 months post-stenting. On the day of surgery, animals were anesthetized as described in the aneurysm creation procedure, and 100 U/kg heparin was administered intra-arterially. Using sterile techniques, the right femoral artery was dissected and ligated at the distal end of the exposure. Just proximal to the ligation, a 6 French (6F) sheath was inserted. A 6F catheter and 0.035-inches guide wire were inserted and advanced under fluoroscopy to the ascending aorta. A DSA of the brachiocephalic artery was acquired using 100% iodinated contrast (Omnipaque 350) and an OEC 980° C-arm fluoroscopic x-ray unit (General Electric). A measurement reference was visible in the field. Once aneurysm formation was confirmed, a microcatheter was then inserted into the guide catheter and navigated into the right subclavian artery over a 0.014-inches microwire. The ReSolv stent was inserted into the hub of the microcatheter, transitioned through the microcatheter and deployed across the aneurysm neck. The microcatheter was removed and a post-stenting DSA was obtained through the guide catheter. Ten minutes post-implantation, another DSA was obtained to document any possible delayed thrombus formation within the stent. Finally, the guide catheter and sheath were removed, and the incision was closed in a multi-layer fashion with 4-0 vicryl sutures. Animals were recovered as described above.

At the follow-up time points, animals were again anesthetized using the technique detailed above. Access to the femoral artery was obtained via cutdown and a 4 French (4F) sheath was inserted. A 4F guide catheter was navigated past the aortic arch over a guidewire and DSA images were obtained. The guide catheter and sheath were removed, the femoral incision was closed, and post-operative analgesia was administered as described above.

### Angiographic analysis

Pre-stenting DSA images were used to measure the parent vessel and aneurysm baseline characteristics, including parent vessel diameter and aneurysm height, width, neck size, and volume. For the remainder of the angiographic analysis, using an online survey, pre- and post-stenting DSA images obtained at the time of final follow-up were presented to a practicing neurointerventionalist from an independent institution who was blinded to follow-up time. Angiographic images were evaluated for safety (in-stent stenosis, parent and jailed side branch patency, stent wall apposition) and efficacy (aneurysm occlusion).

Safety was evaluated in the following manner: in-stent stenosis was classified as mild (0–49%), moderate (50–69%) or severe (70–99%) according to the modified North American Symptomatic Carotid Endarterectomy Trial (NASCET) criteria, with moderate or severe considered significant.^10^ Parent vessel and jailed side branch patency was graded as a 1 (patency with no lumen irregularities), 2 (patency with moderate lumen irregularities), or 3 (patency with severe lumen irregularities) using a simplified system based on the Fitzgibbon patency scale.^11^ Wall apposition was classified as good (stent well apposed along >80% of stented artery), moderate (stent well apposed along 50–80% of the stented artery) or poor (stent well apposed along <50% of the stented artery) using a modified three-point scale based on Rouchaud et al.^12^

The primary efficacy outcome was evaluated by the Raymond–Roy Occlusion Classification (RROC) of grade I (complete obliteration), grade II (residual neck), or grade III (residual aneurysm).^13^ Secondary efficacy outcomes included: 1) the O’Kelly Marotta (OKM) scale which assigns a filling parameter (A-total filling [>95%], B-subtotal filling [5–95%], C-entry remnant [<5%], or D-no filling [0%]) and a stasis parameter (1-no stasis, clearance within the arterial phase prior to the capillary phase, 2-moderate stasis, clearance prior to venous phase, or 3-significant stasis, contrast persists in aneurysm into the venous phase and beyond),^14^ and 2) the 4F Flow diversion Predictive Score (4F-FPS) which assigns a score based on flow-jet of contrast (yes [0 points] vs no [2 points]), residual aneurysm filling (≥ 50% [0 points] vs <50% [1 point]), duration of contrast stasis (arterial or capillary phase [0 points] vs venous phase [1 point]), and aneurysm morphology (fusiform [0 points] vs saccular [1 point]), and has been shown to predict aneurysm occlusion.^15^ Based on previous literature, adequate angiographic occlusion was considered an RROC Grade of 1 or 2, an OKM filling score of C or D, or a 4F-FPS score of 4 or 5.^16–19^

## Results

### Baseline characteristics

One stent was deployed per animal in 13/14 rabbits; the remaining rabbit required two overlapping stents to ensure appropriate aneurysm neck coverage (mean ± SD of 1.08 ± 0.28 stents per animal). Day 0 images to assess baseline characteristics were not available for review in one rabbit due to a technical issue with the fluoroscopy unit. For the remaining rabbits, the mean ± SD parent vessel diameter prior to stenting was 3.50 ± 0.40 mm, aneurysm dimensions were 4.48 ± 1.50 mm in height, 2.63 ± 0.72 mm in width, and 3.18 ± 1.00 mm in neck size, and volume was 19.48 ± 12.69 mm^3^. Angiography follow-up ranged from 1 to 18 months, with a median time of 7.5 months, which is similar to what has been published in previous animal and human studies of flow diversion.

### Safety

Of the 12/14 10-minute post-stenting angiograms that were available for review (2 were not available due to technical issues with the fluoroscopy unit), there was no evidence of delayed thrombus formation within the stent. For the evaluation of safety, follow-up angiograms were available for all 14 rabbits. For all subjects, in-stent stenosis was rated as mild (0–49%), and wall-apposition as good. Parent vessel patency was rated as class 1 in 92.9% (13/14) of cases, with only 7.1% (1/14) displaying patency with moderate lumen irregularities (graded as class 2). All branch vessels (33/33) covered by the stent were patent and were scored as class 1.

### Efficacy

**Figure 1.**
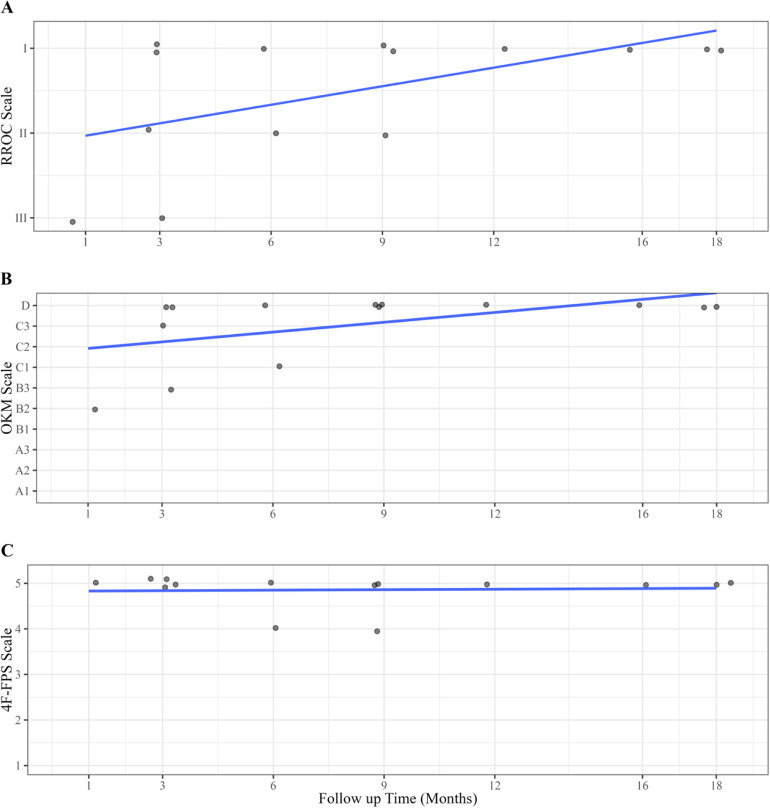
Individual scores on the RROC scale (A), OKM scale (B), and 4F-FPS scale (C) by follow up time (months) with line of best fit (*R*^2 ^= 0.31, 0.28, and <0.01 for RROC, OKM, and 4F-FPS scales, respectively). Each point represents one rabbit at its assigned time point. Data is jittered for individual scores to be visualized.

Follow-up angiograms were available for all 14 rabbits for the evaluation of efficacy. Using the RROC, 64.3% (9/14) of the rabbits were rated as grade I (complete obliteration), 21.4% (3/14) of the rabbits were rated as grade II (residual neck), and 14.3% (2/14) of the rabbits were rated as grade III (residual aneurysm) (Table 1). Based on the primary efficacy outcome measure, adequate angiographic occlusion occurred in 85.7% of cases. Figure 1 provides a detailed breakdown of the RROC results per follow-up group.

**Table 1.** RROC, OKM, and 4F-FPS aneurysm filling evaluation scores for 14 rabbit saccular sidewall aneurysms after deployment of the ReSolv stent at a median follow-up of 7.5 months.

Scale	No. aneurysms (%)
RROC	
I	9 (64.2%)
II	3 (21.4%)
III	2 (14.3%)
OKM	
A: Total Filling, >95%	
1	0 (0%)
2	0 (0%)
3	0 (0%)
B: Subtotal Filling, 5–95%	
1	0 (0%)
2	1 (7.1%)
3	1 (7.1%)
C: Entry Remnant, <5%	
1	2 (14.3%)
2	0 (0%)
3	1 (7.1%)
D: No Filling	9 (64.2%)
4F-FPS	
1	0 (0%)
2	0 (0%)
3	0 (0%)
4	2 (14.3%)
5	12 (85.7%)

On the OKM scale, 64.3% (9/14) of the rabbits were rated as D, 7.1% (1/14) of rabbits were rated as C3, 14.3% (2/14) of the rabbits were rated as C1, 7.1% (1/14) of rabbits were rated as B3, and 7.1% (1/14) of rabbits were rated as B2 (Table 1). On the 4F-FPS scale, 85.7% (12/14) of the rabbits were scored as 5 and 14.3% (2/14) of rabbits were scored as 4. Therefore, 85.7% (12/14) were deemed as having adequate occlusion based on the OKM scale, while 100% (14/14) demonstrated adequate occlusion based on the 4F-FPS scale. Figure 1 provides a detailed breakdown of the efficacy results per follow-up group.

## Discussion

Metal FDSs were developed well after the introduction of coil embolization to address the challenging treatment of wide-necked aneurysms and the higher complication rates of surgical clipping.^20^ In 2011, the pipeline embolization device (PED) (Medtronic, Irvine, CA, USA) was first approved by the FDA with restricted use based on patient age, aneurysm location and aneurysm neck characteristics.^21,22^ The PREMIER trial demonstrated the effectiveness of the PED in 141 patients with 84% of aneurysms achieving a RROC grade of I or II at 12-month follow-up.^23^ Similarly, the Surpass SCENT trial showed an RROC grade I or II in 75.5% of cases, while the flow-redirection intraluminal device (FRED) pivotal study reported a rate of 80% at 12 months.^4,24^

A major issue with commercially available FDSs includes the large metal surface areas required for adequate flow diversion (in the range of 30–35% of total device surface area) and the associated thromboembolic complications that can arise until the stent is fully endothelialized into the blood vessel wall and across the neck of the aneurysm.^25^ In-stent stenosis due to neointimal hyperplasia is also a relevant problem in response to arterial damage caused by stents.^26^ Thrombosis and neointimal hyperplasia can cause vessel narrowing and/or occlusion potentially leading to ischemia or stroke.^27^ The incidence of major or minor stroke after FDS placement ranges between 2% and 12%, while the incidence of significant (>50%) in-stent stenosis at 1 year after placement of commercially available metal FDSs in patients ranges between 2.9% and 4.3%.^4,23,24^ Whether novel stent materials, including bioresorbable polymers, have the potential to reduce these complication rates while remaining equally or more efficacious, remains to be determined.

**Figure 2.**
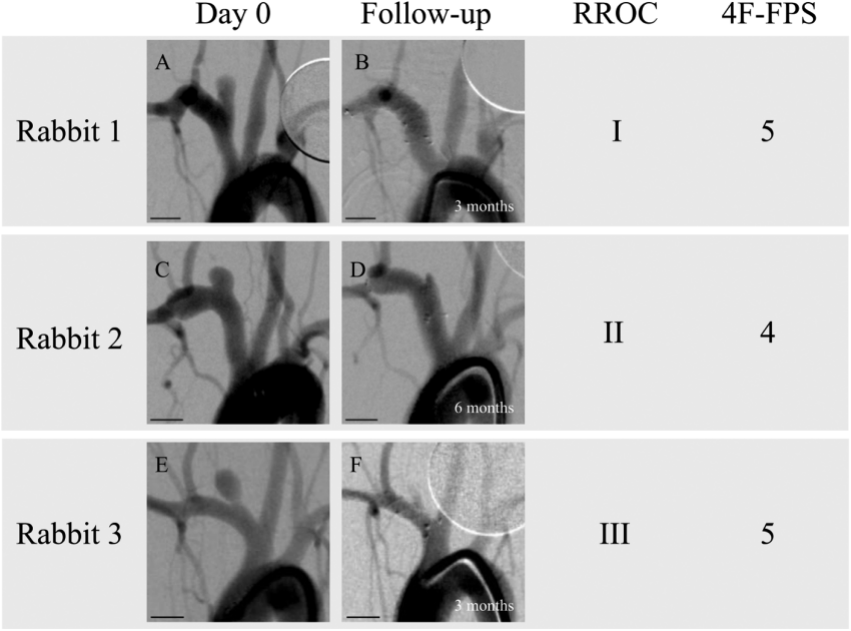
Digital subtraction angiography (DSA) images of three rabbit sidewall aneurysms at treatment (A, C, E) and at follow-up (B, D, F) classified according to the Raymond–Roy and 4F-FPS scoring systems by an independent observer who was blinded to follow-up time points. Scale bars are 5 mm in length. (A) Rabbit 1 at treatment. (B) Rabbit 1 at 3-month follow-up. (C) Rabbit 2 at treatment. (D) Rabbit 2 at 6-month follow-up. (E) Rabbit 3 at treatment. (F) Rabbit 3 at 3-month follow-up.

A previously published report on the novel hybrid polymeric-metal FDS demonstrated immediate flow diversion properties that were comparable to metal stents in vitro and in vivo.^7^ In the present study, we evaluated the medium-term in vivo safety and efficacy of the same stent in a rabbit sidewall aneurysm model in a blinded fashion using an independent observer. Our results demonstrated an adequate occlusion rate of 85.7% as indicated by the primary outcome measure of RROC grade of I or II. Based on the secondary outcome measures of the OKM and 4F-FPS scales, 85.7% and 100% of aneurysms went on to adequately occlude, respectively. Of the two aneurysms that did not adequately occlude based on OKM scale, both were graded as having a 4F-FPS score of 5 and had relatively short angiographic follow-up time points (1 and 3 months). DSA images with corresponding classifications on the RROC and 4F-FPS scales can be visualized in Figure 2. If the predictive nature of the 4F-FPS scores in humans (98% chance of occlusion at mean follow-up of 9 months) translates to this in vivo model, then it suggests that in these two cases, adequate occlusion on OKM scale will likely be reached at a later time point.^15^

The efficacy results of the current study are comparable to what has been observed in pre-clinical trials investigating FDSs composed of metal. Fahed et al. conducted a meta-analysis on aneurysm occlusion following FDS treatment in rabbit elastase aneurysm models, and found an adequate occlusion rate of 79.2% at 1-month post-treatment, 73.5% at 3-month post-treatment, 96.7% at 6-month post-treatment, and 66.7% at 12-month post-treatment.^28^ Pre-clinical investigations of commercially available FDSs such as the PED yielded increased rates of aneurysm occlusion compared to the average adequate occlusion found by Fahed et al.; at a median of 3 months follow-up, a second iteration of the device demonstrated complete occlusion in 94% of cases, and incomplete occlusion in 6% of cases.^29^ These findings are comparable to the results of the current study, which found 85.7% adequate occlusion overall at a median of 7.5 months.

Few other studies on the efficacy of bioabsorbable FDSs in the rabbit model exist in the literature. Nishi et al. published their outcomes on a first prototype 48-strand PLLA biodegradable FDS in 17 rabbit saccular aneurysms.^30^ The stent used in their study, however, required balloon angioplasty after deployment, which was unlike the ReSolv stent. They observed an overall 64.7% (11/17) adequate occlusion (complete occlusion or neck remnant) at a median follow-up time of 3 months.^30^ A more recent investigation by the same group (Sasaki et al.) comparing their second generation PLLA-FDS to a cobalt-chromium FDS found adequate occlusion in 57% and 47%, respectively, at a median follow-up time of 6 months.^31^

In terms of safety, our study demonstrated patency of all parent vessels and jailed side branches, with only one case having moderate lumen irregularities, and no cases of delayed thrombus. Similarly, both Nishi et al. and Sasaki et al. showed 100% patency in all branch vessels, and no downstream arterial occlusion or thrombus formation.^30,31^ Although neither our study, Nishi et al., nor the Sasaki et al. studies followed the animals longer than the time point characterized by initiation of PLLA absorption (on the order of 1.5–2 years depending on exact material properties), the medium-term safety of bioabsorbable stents in rabbit aneurysm models seems to be reasonable.^30,31^ Longer term studies addressing the safety and efficacy of these stents during and after absorption of the polymer component in rabbit models are underway in our laboratory.

There are several limitations of this study, such as the small sample size. The uneven distribution of animals between follow-up time points also limits comparisons between groups and potential trends in aneurysm occlusion rates over time. That said, it is valuable to consider that these study results are much like real-world patient data acquisition that must accommodate varying follow-up timepoints and uneven patient distribution in each group. In addition, the design of the current study was limited in two ways. Firstly, not all animals were terminated at their follow-up time points, which limited a potentially more definitive histological outcome assessment. Secondly, the current study lacked a control group treated by metal FDSs, limiting the ability to directly compare the ReSolv stent and commercially available flow diverters. Finally, this study used qualitative assessment scales to determine aneurysm occlusion status, which is subjective and may affect reliability of these results. However, published aneurysm treatment efficacy scales based on angiography are inherently subjective and used in essentially all reports of outcomes after flow diversion, and the neurointerventionalist interpreting the angiographic data in this study was blinded to the time point of each follow-up, thereby mitigating any potential bias.

In conclusion, the novel polymer and metal hybrid ReSolv stent demonstrates an encouraging angiographic safety profile as well as treatment outcomes in a rabbit sidewall saccular aneurysm model that are comparable to commercially available metal flow diverters. Longer term studies are ongoing to determine the eventual fate of the aneurysm, parent vessel, and jailed side branches after absorption of the polymer component of the stent.

## Abbreviations

CTA: computed tomographic angiography
DSA: digital subtraction angiography
FDS: flow-diverting stent
FRED: flow-redirection endoluminal device
IA: intracranial aneurysm
MRA: magnetic resonance angiography
PED: pipeline embolization device
PLLA: poly-l-lactic acid
RCCA: right common carotid artery
4F: 4 French
6F: 6 French
